# The Activities of Zinc and Magnesium Among Alcohol Dependence Syndrome Patients: A Case-Control Study From a Tertiary Care Teaching Hospital in South India

**DOI:** 10.7759/cureus.24502

**Published:** 2022-04-26

**Authors:** Pratyusha Pavuluri, Sharanya Jangili, Likhitha Ryakam, Sabitha Vadakedath, Sarat Chandan Tummalacharla, Deepthi Kondu, Venkataramana Kandi

**Affiliations:** 1 Biochemistry, RVM Institute of Medical Sciences and Research Center, Siddipet, IND; 2 Psychiatry, RVM Institute of Medical Sciences and Research Center, Siddipet, IND; 3 Medicine, RVM Institute of Medical Sciences and Research Center, Siddipet, IND; 4 Biochemistry, Prathima Institute of Medical Sciences, Karimnagar, IND; 5 Biochemistry, RVM Institute of Medical Sciences Research Centre, Siddipet, IND; 6 Clinical Microbiology, Prathima Institute of Medical Sciences, Karimnagar, IND

**Keywords:** alcohol dependence syndrome, homeostasis, deficiency, magnesium, zinc, microelements

## Abstract

Introduction

Alcohol dependence syndrome (ADS) is a medical condition characterized by regular and excessive consumption of alcohol. ADS is a brain disorder wherein people consume alcohol despite knowing the physical, social, and financial consequences. Zinc and magnesium are microelements that are essential in the proper functioning of human physiological and biological processes. However, the homeostasis of these microelements' is disturbed among ADS patients. The present study aims to assess Zinc and magnesium activities among ADS patients and age-matched controls.

Methods

The study included 100 ADS patients and an equal number of the control group and was conducted between August and September 2021. The study was performed after acquiring approval from the institutional ethics committee. All the study cases were patients attending the department of Psychiatry, RVM Institute of Medical Sciences & Research Centre who were diagnosed with ADS. Both groups used the colorimetric method on ERBA Chem 5+ semi autoanalyzer to estimate serum zinc and magnesium levels.

Results

Among the 100 cases of ADS, 84 (84%) were males, and 16 (16%) were females. Patients were between the age group of 20 and 68 (mean: 42.8 years). Of the 100 controls, 84 (84%) were males, and 16 (16%) were females with a mean age of 43.1 years. The activities of Zinc among ADS patients and the control group were 88.53±18.7 μg/dL and 144.9±38.47 μg/dL (p<0.0001), respectively. The activities of magnesium among the ADS patients and the control group were 1.96±0.46 mg/dL and 2.88±0.4 mg/dL (p<0.0001), respectively.

Conclusions

The activities of Zinc and magnesium have been noted to be significantly lower among ADS patients. Because both these microelements play a vital role in human cells' metabolic and physiologic activities, therapeutic interventions to compensate for such deficiencies while managing ADS patients may prove beneficial.

## Introduction

Alcohol use disorder or alcoholism is a severe problem for public health. People with this disorder consume alcohol very frequently and in excess amounts despite knowing its ill effects [1,2]. Alcohol is a potent substance that causes acute and chronic changes in almost all neurochemical systems and can produce psychological symptoms, including depression, anxiety, and psychosis [3]. An estimate by the National Family Health Survey (NFHS) of India shows that 32% of adult men and <5% of women consume alcohol [4,5]. According to a World Health Organization (WHO) report in 2018, the estimated percentage of people diagnosed with alcohol use disorder in India was 9.1% and 0.5% among males and females. However, people suffering from alcohol dependence syndrome (ADS) were 7% and 0.4% among males and females, respectively [6].

ADS patients experience symptoms that include a strong urge to consume alcohol, difficulties in controlling the urge, physiological withdrawal state when substance use has seized/reduced, evidence of tolerance, progressive neglect of alternative pleasures/interests, and persistent use despite clear evidence of overtly harmful health effects [7]. Despite this, only about 1 in 180 individuals with ADS report getting in-patient treatment/ hospitalization [8]. Alcoholism was previously found associated with magnesium and zinc microelement deficiencies. The cause for this was noted to be multifactorial and included poor nutrition, increased excretion, internal redistribution, and altered transporters [9,10].

Zinc is an essential micronutrient that plays a vital role in cell proliferation, growth, apoptosis, wound healing, and taste sensation. Zinc also enhances the action of insulin, helps the immune system fight off invading microbes, and plays a significant role in major molecular mechanisms such as replication, transcription, translation, gene expression, and gene regulation [11,12]. 

Magnesium is an essential microelement that acts as a cofactor for more than 300 enzymes. It is useful for numerous cellular functions such as skeletal muscle contraction and relaxation, neuromuscular conduction, myocardial contraction, and maintenance of blood pressure [13]. Besides these, magnesium plays a vital role in protein and nucleic acid synthesis, bone mineralization, regulating active transmembrane transport of various cations and anions, and maintaining blood glucose levels [14].

In the present study, we aimed to estimate the activities of Zinc and magnesium in ADS patients and compared them with the control group.

## Materials and methods

This case-control study was performed after acquiring approval from the institutional ethics committee (IEC/RVMIMS&RC/2021/01/04). The study was carried out on patients diagnosed with ADS in the department of Psychiatry, RVM Institute of Medical Sciences & Research Centre, Telangana, South India. The study was done between August and September 2021 and included two groups, cases (100), and an age and sex-matched control group (100).

Inclusion and exclusion criteria

All patients who agreed to provide consent for participation, patients aged more than 18 years, including males and females, and patients diagnosed with ADS were included in the study. People with immunodeficiency disorders, other chronic illnesses, protein-energy malnutrition, pregnant women and lactating mothers, individuals aged less than 18 years, patients already on multivitamins and mineral therapy, occasional alcohol users, and individuals with opiates, amphetamine, tobacco, and other drug dependence were excluded from the study. Three milliliters of venous blood were collected in a plain red vacutainer from all the study participants. Serum zinc and magnesium levels were estimated in both cases and control groups using the colorimetric method on ERBA Chem 5+ semi autoanalyzer. Serum zinc was estimated using Nitro-PAPS (2-(5-nitro-2-pyridylazo)-5-(N-propyl-N-sulfopropylamino) phenol disodium salt dihydrate) that binds with Zinc in an alkaline medium and forms a purple-colored complex which is measured by a spectrometer at 570 nm. Serum Magnesium was estimated using calmagite dye method. Calmagite is a metallo-chromatic indicator that binds with magnesium in an alkaline medium, forms a red-colored complex, and is measured by a spectrometer at 510 to 550 nm. The normal serum zinc and serum magnesium levels were 70-120 μg/dL and 1.7-2.6 mg/dL, respectively.

Statistical analysis

The baseline characteristics of the study participants were entered into Microsoft Excel 2019 and were used for data analysis. The data were presented as percentages, mean and standard deviation (SD), and a student t-test was performed. A p-value < 0.05 was considered statistically significant. SPSS software version 21 (IBM Corp., Armonk, NY) was applied to perform the analyses.

## Results

Among the 100 cases of ADS patients recruited for the study, 84 (84%) were males, and 16 (16%) were females. The patients included were between 20 and 68 years (mean age of 42.8 years). Of the 100 control group patients selected, 84 (84%) were males, and 16 (16%) were females. The control patients were between 22 and 67 years (mean age of 43.1 years).

The activities of Zinc in ADS patients was 88.53±18.7 μg/dL, and in controls, it was 144.9±38.47 μg/dL. A statistically significant variation was observed among cases and controls regarding Zinc (p-value <0.0001). Results also demonstrated that 18% of ADS patients had low serum zinc levels (<70 μg/dL), and the remaining 82% had serum zinc levels within the normal limits (70-120 μg/dL). 

The activities of magnesium in ADS patients was 1.96 ± 0.46 mg/dL, and in controls, it was 2.88±0.4 mg/dL. A statistically significant variation was observed among cases and controls regarding magnesium (p-value <0.0001). Results also revealed that 32% of ADS patients had low serum magnesium levels (<1.7 mg/dL) and remaining 68% had normal serum magnesium levels (1.7-2.6 mg/dL). The details of the serum activities of both Zinc and magnesium among ADS patients and the control group can be viewed in Table 1.

**Table 1:** The activities of serum zinc and magnesium among ADS patients and controls * Statistically significant; ADS: Alcohol dependence syndrome

Subjects/Parameter	Zinc (μg/dL)	Magnesium (mg/dL)
Controls (Mean±SD)	144.9±38.47	2.88±0.40
ADS patients (Mean±SD)	88.53±18.70	1.96±0.46
p-value	<0.0001*	<0.0001*

## Discussion

Alcoholism and ADS are ubiquitous public health-related problems found throughout the world. An ADS patient presents with symptoms that include severe and unstoppable craving for alcohol-containing drinks. ADS patients consume excessive quantities of alcohol and suffer from withdrawal symptoms in the absence of alcohol intake. These patients suffer from social, financial, physical, and psychological imbalances both during the addiction and while undergoing de-addiction treatment [15].

Previous research has shown that ADS patients are predisposed to Zinc (hypozincemia) and magnesium (hypomagnesemia) deficiencies [16-21]. This was attributed to various reasons that include alcohol-induced diuresis (excessive urination) and liver failure resulting in abnormal absorption and transport, among others [22,23]. Because zinc is an essential microelement involved in the metabolism of alcohol, its deficiency may trigger delayed clearance of alcohol in ADS patients. Hypozincemia also results in the lack of the body's ability to repair DNA and causes excessive production of reactive oxygen species (ROS)/free radicals that enhance lipid peroxidation and thus damage the cells [24]. Low zinc activities also cause neuronal damage/death and thereby affect the brain's activities/functions, which could present as hyperexcitability and depression among ADS patients [25]. 

Magnesium deficiency among ADS patients is a consequence of the alcohol's effects on the kidneys and liver and their functions resulting in decreased absorption and transportation [26]. Magnesium deficiency may also be due to reduced dietary intake, malnutrition, and abnormalities in the gastrointestinal functions that present as diarrhea and steatorrhea. Hypomagnesemia predisposes ADS patients to cell damage, cardiac arrhythmias, prothrombotic effects, and convulsions [27]. 

In the present study, the deficiency of Zinc and magnesium was observed in 20-30% of ADS patients. This could be attributed to interference at multiple levels, such as absorption, distribution, metabolism, function, and excretion. Moreover, hypozincemia and hypomagnesemia lead to electrolyte imbalance, enzymatic dysfunction, lipid peroxidation, and cell death. This ultimately affects the brain and results in neurotransmitter dysfunction, defective neuromuscular transmission, neuronal damage, and alterations in neurotransmitter levels. The ADS patients present with symptoms like hyperexcitability due to an imbalance between glutamate and Gamma-aminobutyric acid (GABA) signaling. Changes in the GABA system and mesolimbic pathway can contribute to the anxiogenic and aversive effects of alcohol and lead to relapse for drinking that presents as repetitive and compulsive behavior in ADS patients.

Recently, a few studies reported co-deficiency of Zinc and copper in alcoholic patients who presented with scaly eczematous skin lesions and malaise [28,29]. The patients also showed abnormalities in blood, electrolyte, kidney, and liver function. These reports also suggested that supplementation of both Zinc and copper reversed the skin lesions and organ functions. The role of impaired kidney function in the development of magnesium deficiency among alcohol use disorder patients was recently reported [30]. Such studies impress that alcoholism-induced microelement deficiencies may be more prevalent than previously assumed. Moreover, therapeutic supplementation of the deficient microelements could improve ADS patients' management.

## Conclusions

The serum activities of microelements like Zinc and magnesium are rarely analyzed in people living in low socioeconomic and developing countries like India. However, estimating their activities among ADS patients assumes increased significance because the homeostasis of such microelements is greatly disturbed in these patients. Regular evaluation of the serum activities of essential microelements, including Zinc and magnesium, among ADS patients, can help physicians treating such patients predict and appropriately manage neurological and psychological complications. More evidence-based studies in large groups are required to understand the exact role of these and other essential microelements in developing neuropsychological manifestations related to ADS. Furthermore, research evidence is required to understand the necessity of pharmacological administration of these microelements in treating and managing patients with ADS.

## References

[1] Whiteford HA, Degenhardt L, Rehm J (2013). Global burden of disease attributable to mental and substance use disorders: findings from the Global Burden of Disease Study 2010. Lancet.

[2] Lim SS, Vos T, Flaxman AD (2012). A comparative risk assessment of burden of disease and injury attributable to 67 risk factors and risk factor clusters in 21 regions, 1990-2010: a systematic analysis for the Global Burden of Disease Study 2010. Lancet.

[3] Kurhaluk N (2021). Alcohol and melatonin. Chronobiol Int.

[4] (2022). National Family Health Survey (NFHS-3). https://dhsprogram.com/pubs/pdf/frind3/frind3-vol1andvol2.pdf.

[5] Manimunda SP, Sugunan AP, Thennarasu K, Pandian D, Pesala KS, Benegal V (2017). Alcohol consumption, hazardous drinking, and alcohol dependency among the population of Andaman and Nicobar Islands, India. Indian J Public Health.

[6] (2022). Global status report on alcohol and health 2018. https://www.who.int/publications/i/item/9789241565639.

[7] (2022). The ICD-10 classification of mental and behavioural disorders : clinical descriptions and diagnostic guidelines. https://apps.who.int/iris/handle/10665/37958.

[8] (2022). NDDTC, AIIMS submits report “Magnitude of Substance use in India” to M/O Social Justice & Empowerment. https://pib.gov.in/Pressreleaseshare.aspx?PRID=1565001.

[9] Ham BJ, Choi I-G (2005). Psychiatric implications of nutritional deficiencies in alcoholism. Psychiatr Invest.

[10] Vatsalya V, Kong M, Cave MC (2018). Association of serum zinc with markers of liver injury in very heavy drinking alcohol-dependent patients. J Nutr Biochem.

[11] MacDonald RS (2000). The role of zinc in growth and cell proliferation. J Nutr.

[12] Corniola RS, Tassabehji NM, Hare J, Sharma G, Levenson CW (2008). Zinc deficiency impairs neuronal precursor cell proliferation and induces apoptosis via p53-mediated mechanisms. Brain Res.

[13] Viering DH, de Baaij JH, Walsh SB, Kleta R, Bockenhauer D (2017). Genetic causes of hypomagnesemia, a clinical overview. Pediatr Nephrol.

[14] Gröber U, Schmidt J, Kisters K (2015). Magnesium in prevention and therapy. Nutrients.

[15] Melkonian AJ, Flanagan JC, Calhoun CD, Hogan JN, Back SE (2021). Craving moderates the effects of intranasal oxytocin on anger in response to social stress among veterans with co-occurring posttraumatic stress disorder and alcohol use disorder. J Clin Psychopharmacol.

[16] Stanley PC, Okeke E, Ukoli C (2007). Trace elements profile among alcohol abusers in a Nigerian community. J Appl Sci Environ Manage.

[17] Sullivan JF, Lankford HG (1965). Zinc metabolism and chronic alcoholism. Am J Clin Nutr.

[18] Rodríguez-Moreno F, González-Reimers E, Santolaria-Fernández F (1997). Zinc, copper, manganese, and iron in chronic alcoholic liver disease. Alcohol.

[19] Elisaf M, Bairaktari E, Kalaitzidis R, Siamopoulos KC (1998). Hypomagnesemia in alcoholic patients. Alcohol Clin Exp Res.

[20] Cheungpasitporn W, Thongprayoon C, Qian Q (2015). Dysmagnesemia in hospitalized patients: prevalence and prognostic importance. Mayo Clin Proc.

[21] Vatsalya V, Gala KS, Mishra M (2020). Lower serum magnesium concentrations are associated with specific heavy drinking markers, pro-inflammatory response and early-stage alcohol-associated liver injury. Alcohol Alcohol.

[22] Ragland G (1990). Electrolyte abnormalities in the alcoholic patient. Emerg Med Clin North Am.

[23] Vech RL, Lumeng L, Li TK (1975). Vitamin B6 metabolism in chronic alcohol abuse the effect of ethanol oxidation on hepatic pyridoxal 5'-phosphate metabolism. J Clin Invest.

[24] Topinka J, Binková B, Srám RJ, Fojtíková I (1991). DNA-repair capacity and lipid peroxidation in chronic alcoholics. Mutat Res.

[25] Doboszewska U, Wlaź P, Nowak G, Radziwoń-Zaleska M, Cui R, Młyniec K (2017). Zinc in the monoaminergic theory of depression: its relationship to neural plasticity. Neural Plast.

[26] De Marchi S, Cecchin E, Basile A, Bertotti A, Nardini R, Bartoli E (1993). Renal tubular dysfunction in chronic alcohol abuse--effects of abstinence. N Engl J Med.

[27] Elisaf M, Merkouropoulos M, Tsianos EV, Siamopoulos KC (1995). Pathogenetic mechanisms of hypomagnesemia in alcoholic patients. J Trace Elem Med Biol.

[28] Unhapipatpong C, Warodomwichit D, Chanprapaph K (2019). Alcoholic cirrhosis with zinc and copper co-deficiency. Case Rep Clin Nutr.

[29] Ito H, Ogawa Y, Shimojo N, Kawano S (2022). Copper and zinc deficiency in an alcoholic patient: a case report of a therapeutic dilemma. J Addict Dis.

[30] Vanoni FO, Milani GP, Agostoni C (2021). Magnesium metabolism in chronic alcohol-use disorder: meta-analysis and systematic review. Nutrients.

